# Thermometer Ions, Internal Energies, and In‐Source Fragmentation in Ambient Ionization

**DOI:** 10.1002/mas.21924

**Published:** 2025-01-27

**Authors:** Emilie Bertrand, Valérie Gabelica

**Affiliations:** ^1^ School of Pharmaceutical Sciences University of Geneva Geneva Switzerland

## Abstract

Ionization and fragmentation are at the core of mass spectrometry. But they are not necessarily separated in space, as in‐source fragmentation can also occur. Here, we survey the literature published since our 2005 review on the internal energy and fragmentation in electrospray ionization sources. We present new thermometer molecules to diagnose and quantify source heating, provide tables of recommended threshold (*E*_0_) and appearance energies (*E*_app_) for the survival yield method, and attempt to compare the softness of a variety of ambient pressure ionization sources. The droplet size distribution and desolvation dynamics play a major role: lower average internal energies are obtained when the ions remain protected by a solvation shell and spend less time nakedly exposed to activating conditions in the transfer interface. Methods based on small droplet formation without charging can thus be softer than electrospray. New dielectric barrier discharge sources can gas‐phase ionize small molecules while conferring barely more internal energy than electrospray ionization. However, the tuning of the entire source interface often has an even greater influence on ion internal energies and fragmentation than on the ionization process itself. We hope that this review will facilitate further research to control and standardize in‐source ion activation conditions, and to ensure the transferability of data and research results in mass spectrometry.

## 1 Introduction

It is hard to think of more fundamental subjects in mass spectrometry than ionization mechanisms and ion activation/fragmentation. But these processes are not always decoupled. Soft ionization methods such as electrospray ionization (ESI) (Yamashita and Fenn 1984) and matrix‐assisted laser desorption/ionization (MALDI) (Karas et al. 1987) revolutionized mass spectrometry, notably because they were much softer than electron ionization (EI) and chemical ionization (CI). But although intact molecular ions are effortlessly produced by ESI and MALDI, ions just emitted from ESI or MALDI are hotter than the ambient temperature (~500–600 K for ESI (Carpenter et al. 2017; Drahos et al. 1999; Naban‐Maillet et al. 2005), ~800–1000 K for MALDI (Gabelica, Schulz, and Karas 2004b; Schulz et al. 2006)). More importantly, for atmospheric pressure ionization, the transfer interface modulates this ion internal energy further, through collisional activation/deactivation processes that depend on source pressure, hardware geometry and voltages applied. Thus, using ESI does not guarantee against in‐source activation and in‐source fragmentation.

Analytical chemists now come to realize this the hard way: Siuzdak and collaborators claim that, in metabolomics, in‐source fragments make up over 70% of ions selected for automatic MS/MS and interpreted as if they were intact molecular ions (Giera et al. 2024). This causes massive problems for annotation and quantification. All molecular classes are concerned: metabolites (Xu, Lu, and Rabinowitz 2015), and particularly lipids (Criscuolo, Zeller, and Fedorova 2020; Gathungu et al. 2018; Hu et al. 2022), nucleosides (Chen et al. 2023b), N‐glycans (Liew, Chen, and Ni 2022), modified amino acids (Mamani‐Huanca et al. 2020), but also organic pollutants (Xie et al. 2023) and natural compounds (Chen et al. 2023a). These recent references illustrate a pressing analytical problem. Internal energy qualification and standardization can be the solution.

The present festschrift volume honoring Prof. Zenobi was the perfect occasion to summarize the literature that appeared since our 2005 review of internal energy and fragmentation in electrospray ionization sources (Gabelica and Pauw 2005). Studying ion internal energy as a function of the ionization method and tuning parameters was often considered a niche fundamental topic, but there was a revival of interest in using the survival yield of thermometer ions to compare ion internal energies obtained from the variety of atmospheric pressure ionization methods developed over the last 20 years. The Zenobi group was very active in developing alternative ambient ionization sources and characterizing their softness through internal energy distributions (Gyr et al. 2019; Huba, Mirabelli, and Zenobi 2019; Kaeslin et al. 2022; Nudnova, Zhu, and Zenobi 2012; Schmitz et al. 2008; Touboul, Jecklin, and Zenobi 2008). Here we will systematically compare internal energy distributions obtained with a wide variety of ambient ionization sources. However, we will not cover all recent developments in desorption/ionization sources, or ion activation in MS/MS experiments.

The first attempts to quantify ion internal energy in ion sources used ions with sequential neutral losses (e.g., CO losses from ferrous complexes) (Cooks et al. 1990; DeKrey et al. 1986; Hand, Majumdar, and Graham Cooks 1990; Kenttämaa and Cooks 1985). Later, De Pauw proposed instead to use series of Benzylpyridinium ions, each fragmenting via pyridine loss, with barriers depending on the substituent (Collette and De Pauw 1998; De Pauw et al. 1990; Derwa and Pauw 1989; Derwa, De Pauw, and Natalis 1991). During the last 20 years, several classes of thermometer ions, i.e., ions used to quantify the internal energy imparted to them, were also developed as complements to the benzylpyridinium salts. Benzylammonium ions are useful to characterize ambient ionization methods that require volatile analytes and not salts (Stephens et al. 2015). We also survey these new thermometer molecules and gathered the highest quality values of their bond dissociation energies and threshold energies for the appearance of the fragments.

**Figure 1:**
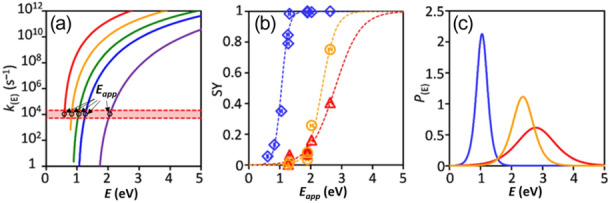
Illustration of the survival yield method (adapted from a figure reproduced with permission from Asakawa D, Yamamoto R, Hanari N, Saikusa K. Differences in the internal energies of ions in electrospray ionization mass spectrometers equipped with capillary‐skimmer and capillary‐RF lens interfaces. Anal Meth 2023b;15(45):6150‐6158. Copyright {2023} RSC Publishing) (Asakawa et al. 2023b). (a) Rate constants *k* for the dissociation of five benzylammonium thermometer ions (‐BnNH_3_^+^) C_9_H_8_N‐CH_2_NH_3_^+^ (red), C_8_H_6_N‐CH_2_NH_3_^+^ (orange), αCH_3_, CH_3_O‐BnNH_3_^+^ (green), CH_3_O‐BnNH_3_^+^ (blue), and BnNH_3_^+^ (purple), as a function of internal energy *E*. The horizontal dashed band indicates the 1/τ value; *k*(E) and *τ* are used to determine the appearance energy *E*_app_ for each thermometer ion. (b) experimental SY values obtained on three different source interfaces (TSQ Fortis Plus in blue, TSQ Quantis Plus in orange and TSQ Altis Plus in red), as a function of the *E*_app_ values of thermometer ions. The dashed lines indicate fitting to sigmoidal curves. (c) Internal energy distributions *P*(*E*) obtained from the derivative of the sigmoidal curve in (b). The assumptions are that all thermometer ions have exactly the same internal energy, and that dissociation is complete if *E* > *E*_app_ and absent if *E* < *E*_app_. [Color figure can be viewed at wileyonlinelibrary.com]

The methods to determine or model internal energy distributions were already established 20 years ago, and there was no progress on these fundamental aspects besides a re‐evaluation of the threshold energies. The reader is encouraged to consult sections IV and VI.A and VI.B of our past review for a detailed tutorial on the theoretical aspects (Gabelica and Pauw 2005). The key definitions and concepts can be summarized as follows:

1. **Thermometer ions** are ions for which we know the **threshold dissociation energy** ***E***_**0**_, which is the minimum energy required for dissociation to occur. Each selected thermometer ion has a different *E*_0_. *E*_0_ values can be either calculated, or measured with guided ion beam mass spectrometry (GIBMS) (Armentrout 2002). *E*_0_ is easiest to calculate for a simple bond cleavage leading to a neutral loss. Quantum chemical calculations provide *E*_0_ values.
2. **Kinetic shift.** If we would impart an amount of energy just equal to *E*_0_, the ions would require a much longer time to fragment than the time the ions take to travel from the source to the mass analyzer. This is because the total internal energy *E* is redistributed among all vibrational **degrees of freedom** of the ion (*N* = number of atoms; ***DOF*** = **3** ***N*** **‐6**), while only a single combination of vibrations leads to dissociation. It is hard to estimate the ion residence time; in most electrospray sources, a crude estimation is that the **time scale for dissociation** ***τ*** = 10^−4 ^s. The kinetic shift (*ks*) is the difference between *E*_0_ and the actual **appearance energy** ***E***_**app**_ (*E*_app_ = *E*_0_ + *ks*), the energy above which the **dissociation rate constant** ***k*** is equal to 1/*τ*.
3. **The Rise‐Ramsperger‐Kassel‐Marcus (RRKM) theory** is a probabilistic model allowing to calculate ***k*** **(** ***E*** **)**, i.e., how the dissociation rate *k* depends on the **internal energy** ***E*** (Figure 1a). RRKM calculations require knowing the threshold *E*_0_, but also the vibrational frequencies of the precursor ion (this is easy to calculate and can be measured in limited cases) and the vibrational frequencies of the transition state (this harder to calculate (Rodgers, Ervin, and Armentrout 1997) and cannot be measured). RRKM calculations are required to determine *E*_app_.
4. **Internal energy distributions** ***P*** **(** ***E*** **):** not all ions have the same internal energy *E*. An ion population has an internal energy distribution: a distribution of probabilities to have an energy *E*. **<** ***E***_**int**_ **> is the average energy of the internal energy distribution** ***P*** **(** ***E*** **).** The most common type of internal energy distribution is the Boltzmann distribution, to which a temperature can be associated. Ideally, the internal energy distribution should be a symmetrical Boltzmann distribution but in practice, with different environments, pressure and sources, the shape of the distribution can deviate from the Boltzmann distribution.
5. **The survival yield method** (Figure 1) is the simplest way to determine the internal energy distribution: the survival yield (**SY** = fraction of intact precursor ion) is measured for a set of thermometer ions with similar DOF but different *E*_app_. For each experimental condition, the SY of each molecule is plotted as a function of *E*_app_ (Figure 1b). The first derivative gives *P*(*E*) (Figure 1c). Given that the *x*‐axis of Figure 1b is *E*_app_, assuming that this derivative represents *P*(*E*) means that one is assuming that all thermometer ions have exactly the same internal energy, and that the dissociation is 100% complete if *E* > *E*_app_ and absent if *E* < *E*_app_ (for fuller discussion of the assumptions, see (Gabelica and Pauw 2005). Many groups used *E*_0_ values instead of *E*_app_ values, because *E*_app_ would require RRKM calculations and knowing *τ*. With this simplification, the internal energies are systematically underestimated, but comparisons between different sources or conditions on the same instrument can still be made. Moreover, the SY curve is fitted by a sigmoid and this regression function is derived to give *P*(*E*). However, fitting by a symmetric sigmoid will automatically give a symmetric *P*(*E*). Revealing the asymmetry in *P*(*E*) requires using asymmetric sigmoid functions and having enough data to do so (Gabelica, De Pauw, and Karas 2004a). In the last 20 years, everyone used symmetric sigmoids, but note that this assumption is not justified when internal energy distributions deviate from the Boltzmann distribution.
6. The **characteristic temperature (** ***T***_**char**_ **)** of an ion is the temperature of the Boltzmann distribution that would give the same survival yield as the experimental one (Drahos et al. 1999; Drahos and Vékey 1999). In contrast to the survival yield method, the characteristic temperature can be calculated for each thermometer ion. Characteristic temperatures obtained for a series of thermometer ions having the same number of DOF usually agree. *T*_char_ can be obtained by programming the RRKM and *P*(*E*) functions. The **MassKinetics software** includes the *T*_char_ determination, but also more advanced master equation modeling of internal energy distributions, adapted to MS/MS (Drahos and Vékey 2001).

## 2 Thermometer Ions

### 2.1 Benzylpyridinium Ions and Their Derivatives

#### 2.1.1 Fragmentation Pathways of Benzylpyridinium Ions

Benzylpyridinium ions were initially chosen for internal energy distribution determinations because the C‐N simple bond cleavage, resulting in a benzylium ion and pyridine, is the predominant fragmentation reaction (Collette and De Pauw 1998). Several studies focused on other fragmentation pathways of benzylpyridiniums, and how they should be taken into account in the survival yield method.

**Figure 2:**
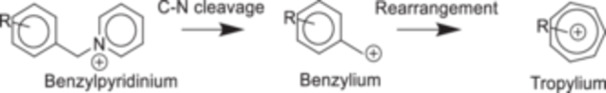
Main fragmentation pathway of benzylpyridinium ions.

One question was whether the predominant fragment ion was a benzylium ion as initially assumed, or a tropylium ion, which would entail a rearrangement (Figure 2). Pieces of evidence for tropylium are based on kinetic energy release experiments (Zins et al. 2009a) and gas‐phase reactivity of the fragment ion (Zins, Pepe, and Schröder 2010; Zins et al. 2009b). The benzylium ion can indeed make adducts with solvent molecules such as acetonitrile (Gabelica et al. 2001; Lecchi et al. 2009; Morsa et al. 2020a). If this happens, for the survival yield method the adducts peaks must be summed with their respective fragments. Infrared multiple photon dissociation (IRMPD) spectroscopy demonstrates that the formation of benzylium or tropylium depends of the activation regime (Morsa et al. 2014b). In summary, the benzylium ion forms first, and rearrangement into a tropylium ion occurs subsequently, if the reaction time and collision energy are sufficient. Thus, even if a fraction of the fragment ion is in the tropylium form when arriving at the detector, the rate‐limiting step to form the fragment ion remains the simple bond cleavage, the relevant transition state is as assumed in the initial concept, and the calculated bond dissociation energies are correct when assuming the simple bond cleavage.

**Figure 3:**
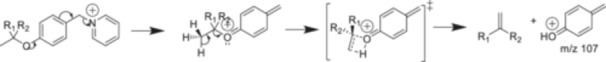
Internal elimination mechanism of Benzhylpyridinium species (with R_1_ = R_2_ = H, R_1_ = H,R_2_ = Me or R_1_ = R_2_ = Me).

Besides, benzylpyridinium ions such as *para*‐ethoxybenzylpyridinium (*p*‐OEt‐Bz), *para*‐isopropoxybenzylpyridinium (*p*‐OiPr‐Bz) or *para*‐*tert*‐butoxybenzylpyridinium (*p*‐O*t*Bu‐Bz) can form other fragments than the expected benzylium ion (Ieritano and Hopkins 2021). As shown in Figure 3, this unexpected fragment results from an intermolecular elimination formed via a four‐membered transition state.

**Figure 4:**
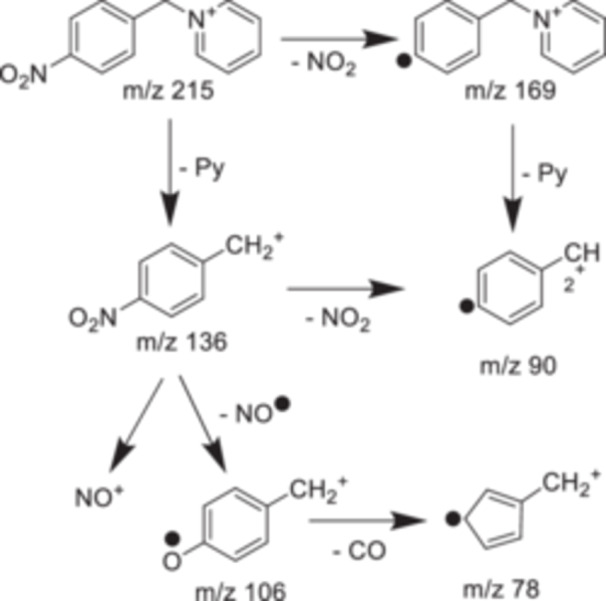
Detailed fragmentation pathway of p‐nitrobenzylpyridinium.

Alternative fragmentation pathways of *p*‐nitrobenzylpyridinium are most prominent. A degradation of the nitro group can be observed in similarly energetic conditions as the N‒C bond cleavage (Asakawa and Saikusa 2023; Barylyuk et al. 2010; Carpenter et al. 2017) (Figure 4). Barylyuk et al. listed all the fragments of benzylpyridinium ions observed in CID experiments (Barylyuk et al. 2010) (Table 1). They compared internal energy calculation performed either by considering only the direct bond cleavage between C and N, or by summing up all fragment ion intensities. The presence of additional fragments influences the ion internal energy calculations, especially at higher internal energies where the alternatives dissociation mechanisms become predominant. The consecutive fragmentation pathways of the *p*‐NO_2_ BzPy were also studied by guided ion beam MS/MS (Carpenter et al. 2017), and the energy parsing validates the approach of summing the fragments ion intensities at *m/z* = 106, 90 and 78 with the benzylium fragment, while the *m/z* 169 signal should be summed with the precursor.

**Table 1:** Benzylpyridinium fragments identified in CID experiments, comparison of traditionally used E_0_ values (AM1) with experimental ones obtained by guided ion beam mass spectrometry (GIBMS) or calculated at high level of theory (MP2(full)).

Substituent			Additional fragments^a^					
	*m/z* BzPy^+^	*m/z* Bz^+^	*m/z*	*Formula*	*E*_0_ (eV) AM1^b^	*E*_0_ (eV) MP2(full)^c^	*E0 (eV) CCSD(T)* c	*E*_0_ (eV) GIBMS exp^d^
*p*‐NO_2_	215	136	169	C_12_H_11_N	2.35	2.79	2.84	3.04 ± 0.12
			106	C_7_H_6_O				
			90	C_7_H_6_				
			78	C_6_H_6_				
*p*‐CN	195	116	89	C_7_H_5_	2.10	/		/
*p*‐H	/	/	/	/		2.48	2.50	2.58 ± 0.15
*p*‐F	188	109	89	C_7_H_5_	1.87			
			83	C_5_H_4_F				
*p*‐Cl	204	125	99	C_5_H_4_Cl	1.90	/		/
			89	C_7_H_5_				
*p*‐CH_3_	184	105	103	C_8_H_7_	1.77	2.26	2.27	2.26 ± 0.13
			79	C_6_H_7_				
			77	C_6_H_5_				
*p*‐OCH_3_	200	121	106	C_7_H_6_O	1.51	1.86	1.84	1.93 ± 0.08
			91	C_7_H_7_				
			77	C_6_H_5_				

^a^ Barylyuk et al. (2010).

^b^ Gabelica, De Pauw, and Karas (2004a).

^c^ DeBord et al. (2013).

^d^ Carpenter et al. (2017).

**Figure 5:**
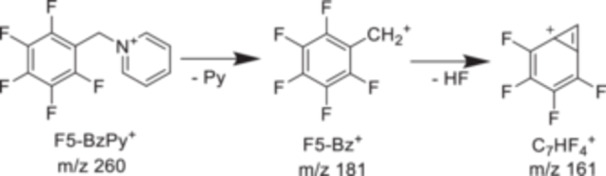
Fragmentation pathway of pentafluorobenzylpyridinium ions.

As an alternative to the *p*‐NO_2_ benzylpyridinium, Asakawa and collaborators searched for a benzylpyridinium ion with a high dissociation threshold but less secondary fragmentation. They propose pentafluorobenzylpyridinium, with an *E*_0_ value of 2.95 eV calculated at the CCSD(T)/6‐311 + + G(d,p)//M06‐2X‐D3/6‐311 + + G(d,p) level of theory. Its dissociation mechanism is shown in Figure 5. The C_7_HF_4_^+^ fragment observed at *m/z* 161 is minor compared to the F_5_‐Bz^+^ fragment and should be summed up with the latter. It does not introduce errors in the survival yield values.

#### 2.1.2 New *E*_0_ and *E*_app_ Values for Benzylpyridinium Ions

One difficulty in estimating the internal energy distribution or internal temperature quantitatively is to estimate the bond dissociation energies (*E*_0_) and kinetic shifts. Until 2017, only calculated (predicted) values were available. As the computational cost of such calculations decreased over the years, values at higher levels of theory and with larger basis sets (therefore, more accurate values) were obtained. DeBord and collaborators showed how the *E*_0_ values converge as the level of theory increases, and published a very complete set of *E*_0_ values at the CCSD(T) level with a 6‐311 + + G(d,p) basis set (geometry optimization at the B3LYP level with a 6‐311 + + G(2 d,p) basis set) (DeBord et al. 2013).

There were also attempts to determine *E*_0_ values experimentally (Gatineau et al. 2017). The most accurate first‐principles experimental determination of *E*_0_ values and activation entropy were obtained using guided ion beam tandem mass spectrometer (GIBMS, results in Table 1) (Carpenter et al. 2017). The four measured *E*_0_ values agree with the calculations of DeBord, and with *E*_0_ calculations at the MP2(full)/6‐311 + G(2 d,2p) level with correlation of all electrons and with counterpoise correction for the basis set superposition error (Carpenter et al. 2017). The GIBMS study confirms that the hypothesis of the loose transition state is valid. In its supporting information, the same paper also provides frequency sets for the precursor ion and for the transition state of *p*‐OCH_3_, *p*‐CH_3_, *p*‐H and *p*‐NO_2_ benzylpyridiniums that match the activation entropies but are derived from a variational study and not by a rough scaling of the precursor ion frequencies. These frequency lists are precious for RRKM calculations, which are necessary to calculate kinetic shifts, use the characteristic temperature method, or for any type of master equation modelling.

An implication of these new studies is that, when comparing internal energies or effective temperatures determined using benzylpyridinium ions over the years, it is important to scrutinize which *E*_0_ values were used. Values obtained at a lower level of theory are usually lower, meaning that internal energies were underestimated in older publications. Upon reviewing articles that have used the survival yield method with benzylpyridiniums to study internal energy in sources, we found that most were using *E*_0_ values calculated at the AM1 level in 2004, and only few recalculated *E*_0_ values at high level (typically, CCSD(T)). Also, very few studies included the kinetic shift effect, further underestimating the internal energies. Table 2 summarizes the most accurate, recommended *E*_0_ values (calculated at the CCSD(T) level) for a large set of benzylpyridinium ions.

**Table 2:** Recommended *E*_0_ values for benzylpyridinium ions.

Benzyl substituent	*E*_0_ (eV), CCSD(T)	*E*_*app*_ *(eV)* for 10^−5^ < τ < 10^−4^ s^b^	*E*_*app*_ *(eV)* for τ = 10^−4^s^c^, ^d^
p‐OCH_3_	1.840^a^	2.61 ± 0.09	2.47 ± 0.15
p‐OH	1.992^a^		
p‐tBu	2.194^a^		
p‐CH_3_	2.267^a^	3.37 ± 0.12	3.05 ± 0.26
p‐Br	2.371^a^		
p‐Cl	2.375^a^		
p‐F	2.392^a^		
o‐CH_3_	2.393^a^		
m‐CH_3_	2.417^a^		
m‐OCH_3_	2.491^a^		
‐H	2.500^a^	3.59 ± 0.12	3.46 ± 0.30
m‐F	2.668^a^		
p‐CN	2.736^a^		
p‐CF_3_	2.74^b^	4.61 ± 0.18	
m‐CN	2.794^a^		
p‐NO_2_	2.843^a^	4.66 ± 0.18	4.68 ± 0.29
1,2,3,4,5‐F	2.95^b^	4.89 ± 0.16	
3,5‐NO_2_	3.133^a^		

^a^ DeBord et al. (2013).

^b^ Asakawa and Saikusa (2023).

^c^ Carpenter et al. (2017).

^d^ For *E*_app_ values at τ = 2 × 10^−4^ s and 5 × 10^−4^, see Rahrt et al. (2019).

#### 2.1.3 Substituted Benzhydrylpyridinium Ions (R,R'‐Bhpy^+^)

**Figure 6:**
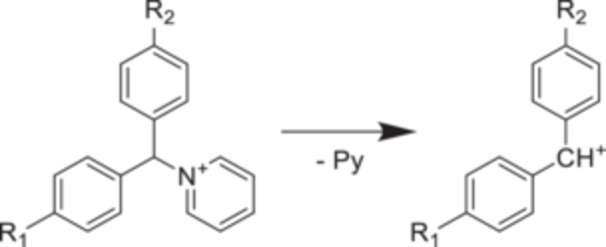
Chemical structure and fragmentation pathway of the substituted benzhydrylpyridiniuim ions (Adapted with permission from Rahrt R, Auth T, Demireva M, Armentrout PB, Koszinowski K. Benzhydrylpyridinium Ions: A New Class of Thermometer Ions for the Characterization of Electrospray‐Ionization Mass Spectrometers. Anal Chem. 2019;91(18):11703–11711. Copyright {2019} American Chemical Society.) (Rahrt et al. 2019).

To lower *E*_0_, the key idea was to add a second aryl group to benzylpyridiniums to weaken the C‐N bond (Figure 6) (Rahrt et al. 2019). Quantum chemical calculations at the DLPNO‐CCSD(T)/CBS//PBE0‐D3BJ level give *E*_0_ between 0.70 and 1.74 eV (Table 3). The calculated value were confirmed by GIBMS. The dissociation energy is lower because the fragment is more stable than the classical R‐BnPy^+^. The study also provides appearance energies, considering the kinetic shift at different time scales.

**Table 3:** Calculated (DLPNO‐CCSD(T)/CBS//PBE0‐D3BJ) and experimental (guided ion beam mass spectrometry, GIBMS) dissociation energies E_0_ for the pyridine loss of benzhydrylpyridinium ions (Rahrt et al. 2019).

R_1_,R_2_‐BhPy	*E*_0_ (eV) CCSD(T)	*E*_app_ (eV) CCSD(T)	*E*_0_ (eV) GIBMS	*E*_app_ (eV) GIBMS
H, H	1.74	2.31	1.79 ± 0.10	2.43 ± 0.24
Me, Me	1.52	2.05	1.55 ± 0.13	2.16 ± 0.29
H, OMe	1.41	1.76	1.37 ± 0.14	1.70 ± 0.27
Me, OMe	1.33	1.70		
OMe, OMe	1.18	1.47		
NPh_2_, NPh_2_	0.70	0.97		

*Note:* Appearance energies are given for a time constant of 10−4 s; values for values at τ = 2 × 10^−4^ s and 5 × 10^−4^, see Rahrt et al. (2019).

#### 2.1.4 Benzylpyridinium‐Substitued Porphyrins

**Figure 7:**
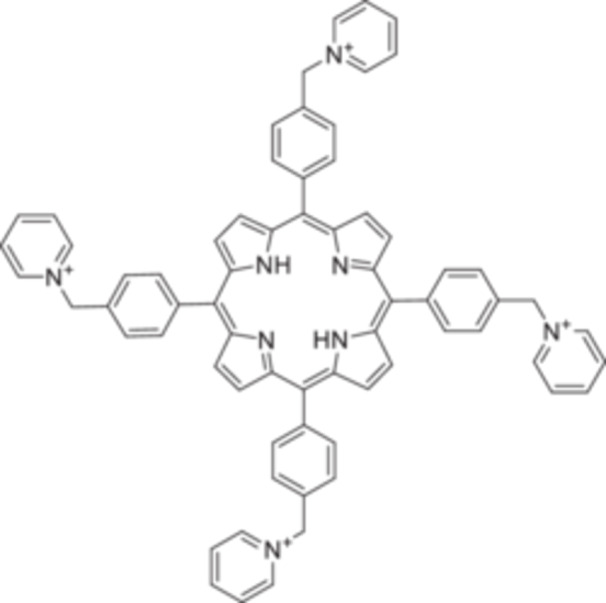
Benzylpyridinium‐subsitituted derivative H_2_P^4+^.

In 2017, the Kappes group introduced benzylpyridinium‐substituted porphyrins (cationized or not) as higher‐mass, multiply charged thermometer ions (Brendle et al. 2017). The H_2_P^4+^ derivative (Figure 7) has 384 degrees of freedom. They fragment by the successive losses of pyridine molecules. The bond dissociation energies calculated using the BP86 functional and def‐SVP basis set range between 1.30 eV (HP^3+^, first pyridine loss) to 3.11 eV (H_2_P^4+^, third pyridine loss). The molecules were used to evaluate bond dissociation energies in a linear ion trap, but to date, they were not used to evaluate internal energies in ionization sources.

### 2.2 New Classes of Thermometers Ions

Critical energy values of benzylpyridinium ions are still rather high and do not always allow for reconstructing a nice survival yield curve in the softest ion production and ion transfer conditions. Several groups proposed new classes of thermometer ions with lower threshold energies, yet with the same advantages as the benzylpyridinium ions, such as a predominant simple bond cleavage fragmentation pathway, and a set of substituted molecules with similar masses, structures and numbers of degrees of freedom.

#### 2.2.1 Protonated Esters

**Figure 8:**
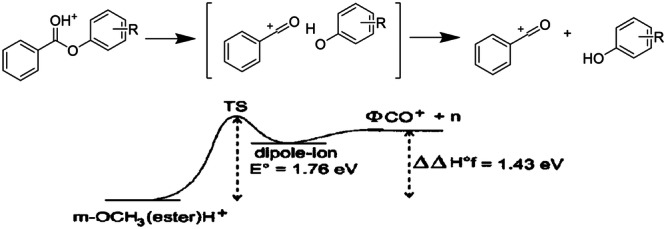
Proposed fragmentation pathway of protonated esters (Adapted with permission from Naban‐Maillet J, Lesage D, Bossee A, et al. Internal energy distribution in electrospray ionization. *J Mass Spectrom*. 2005;40(1):1‐8. Copyright {2005} John Wiley and Sons.) (Naban‐Maillet et al. 2005).

Besides benzylpyridinium ions, the Tabet group proposed a series of protonated esters, shown in Figure 8 (Naban‐Maillet et al. 2005). The *E*_0_ energies defined as shown on the figure are lower than with benzylpyridinium ions with the corresponding substituents. However, fragmentation proceeds through a rearrangement, which makes it harder to define the transition state and therefore to calculate accurate *E*_0_ values, let alone the kinetic shifts which require the full RRKM curves. Without a confirmation of the proposed values by GIBMS, we do not recommend using protonated esters as thermometer ions for quantitative purposes. They can still be used for comparisons.

#### 2.2.2 Phenethylamines

Asakawa and collaborators published several reports on the in‐source fragmentation of protonated phenylethylamine metabolites (Asakawa et al. 2020; Asakawa and Saikusa 2021; Asakawa et al. 2021b), as well as tryptophan‐ (Asakawa et al. 2021a) or histidine‐derived metabolites (Asakawa, Todoroki, and Mizuno 2022). Several protonated ions of these families lose a small neutral molecule (NH_3_, H_2_O,…) in ESI source conditions typically used in metabolomics studies, and are more fragile than the p‐OCH_3_ benzylpyridinium ion. An extensive study of 15 molecules established the *E*_0_ values at the highest level of theory possible (CCSD(T)/cc‐pVTZ//MP2(full)/6‐311 + + G(d,p), see Table 4 (Asakawa and Saikusa 2021)), then tested numerous density functionals to delineate which of these less computationally expensive methods would give the best calculated survival yield values (this requires both accurate *E*_0_ and good *k*(*E*) functions, and thus a good description of the transition states). The authors conclude that the B3LYP functional (the most traditionally used by chemists) underestimates *E*_0_ values. In contrast, wB97‐XD, M06‐2X‐D3, MN11, MN12‐SX and MN15 give values close to CCSD(T) for a fraction of the computational cost. Double hybrid functionals were also reliable.

**Table 4:** Bond dissociation energies or fragmentation thresholds for fragile metabolites (Asakawa and Saikusa 2021).

Molecule	Fragment	*E*_0_ (eV) CCSD(T)
Octopamine	[M + H ‐ H_2_O]^+^	1.05
Noradrenaline	[M + H ‐ H_2_O]^+^	1.08
2‐Amino‐1‐phenylethanol	[M + H ‐ H_2_O]^+^	1.32
Synephrine	[M + H ‐ H_2_O]^+^	1.31
Adrenaline	[M + H ‐ H_2_O]^+^	1.36
4‐Methoxyphenethylamine	[M + H ‐ NH_3_]^+^	1.32
Tyramine	[M + H ‐ NH_3_]^+^	1.42
Dopamine	[M + H ‐ NH_3_]^+^	1.49
4‐Methylphenethylamine	[M + H ‐ NH_3_]^+^	1.63
4‐Fluorophenethylamine	[M + H ‐ NH_3_]^+^	1.70
4‐Chlorophenethylamine	[M + H ‐ NH_3_]^+^	1.71
N‐Methyltyramine	[M + H ‐ CH_3_N]^+^	1.88
N,N‐Dimethyltyramine	[M + H ‐ C_2_H_7_N]^+^	2.19
4‐Trifluorophenethylamine	[M + H ‐ NH_3_]^+^	1.97
4‐Nitrophenethylamine	[M + H ‐ NH_3_]^+^	2.05

#### 2.2.3 Substituted Benzylammonium Ions

**Figure 9:**
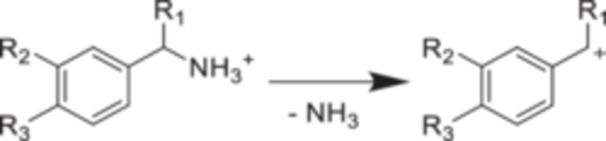
Structure and fragmentation of benzylammonium ions.

The fragmentation of ammonium‐containing metabolites (Chai et al. 2012) also inspired the development of a series of benzylammonium thermometer ions, with different benzyl substituents. These thermometer ions were more adequate than benzylpyridinium salts to characterize the internal energies of ions generated by Low Temperature Plasma (LTP) and Atmospheric Pressure Chemical Ionization (APCI) (Stephens et al. 2015), which require sufficient analyte volatility. The activation of benzylammonium species leads to the formation of a benzyl cation via C‐N cleavage (Figure 9). Calculations at the CCSD(T)/cc‐PVTZ//M06‐2X‐D3/6‐311 + + G(d,p) level of theory give their dissociation energies (Table 5). As detailed further in Section 4, benzylammonium ions have been adopted widely to estimate the internal energies of ions in other sources than the ESI (Bouza et al. 2023; Dumlao, Khairallah, and Donald 2017a; Kaeslin et al. 2022; Stephens et al. 2015). Asakawa and Saikusa showed that benzylammoniums and benzylpyridiniums could be combined in the survival yield method (using *E*_app_ values for both) to reconstruct a single survival yield sigmoidal curves (Asakawa and Saikusa 2022).

**Table 5:** Masses, dissociation energies E_0_ and appearance energies E_app_ of substituted benzylammonium (Bn) and benzhydrylammonium (Bh).

Abbreviation	R_1_	R_2_	R_3_	*m/z*		E_0_ (eV) B3LYP/BS^a^, *	E_0_ (eV) CCSD(T)^b^	E_app_ (eV) CCSD(T) at τ = 10^−4^ s^b^
				*Precursor ion*	*Fragment ion*			
Me, OMe‐BnNH_3_^+^	CH_3_	H	OCH_3_	152.11	135.08		0.89	1.05
H, H‐BhNH_3_^+^	C_6_H_5_	H	H	184.11	167.09		1.07	1.26
OMe‐BnNH_3_^+^	H	H	OCH_3_	138.09	121.06	1.10	1.06	1.25
CH_2_O_2_‐BnNH_3_^+^	H	CH_2_O_2_	H	152.07	135.04		1.10	1.31
Me‐BnNH_3_^+^	H	H	CH_3_	122.10	105.07	1.45	1.48	1.89
Cl‐BnNH_3_^+^	H	H	Cl	142.04	125.02	1.58	1.57	1.98
F‐BnNH_3_^+^	H	H	F	126.08	109.04	1.58	1.60	1.82
H‐BnNH_3_^+^	H	H	H	108.08	91.05	1.69	1.71	2.02
CF_3_‐BnNH_3_^+^	H	H	CF_3_	176.07	159.04	1.91	1.89	2.62
NO_2_‐BnNH_3_^+^	H	H	N0_2_	153.07	136.04	2.04	2.00	2.80

^a^ Stephens et al. (2015).

^b^ Asakawa and Saikusa (2022).

* BS = Broken Symmetry.

#### 2.2.4 Phenyl Sulfate Derivatives

**Figure 10:**
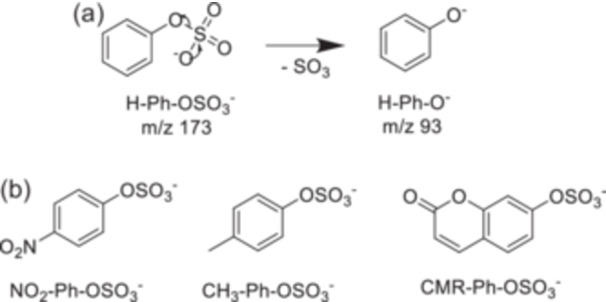
(a) Fragmentation pathway of the H‐Ph‐OSO_3_^−^ and (b) structure of the other phenyl sulfate derivatives.

These thermometer ions were developed to characterize the internal energy distribution in negative ion mode (Asakawa 2023a). Figure 10a presents the structure of the phenyl sulfate derivatives ions. The fragmentation pathway of H‐Ph‐OSO_3_^−^ is shown in Figure 10b the phenolate anions are produced from the phenyl sulfates by simple S‐O bond dissociation and formation of sulfur trioxide. The *E*_0_ values of phenyl sulfate derivatives, calculated by CCSD(T)/6‐311 + + G(2df,p)//M06‐2X‐D3/6‐311 + + G(d,p) level of theory, are gathered in Table 6. Frequencies for RRKM calculates are provided in the article's supporting information (Asakawa 2023a).

**Table 6:** Dissociation energies E_0_ values of phenyl sulfate thermometer ions for the negative mode (Asakawa 2023a).

Phenyl Sulfate derivatives	*E*_0_ (eV) CCSD(T)	*E*_app_ (eV) (τ = 125 ± 75 µs)
H‐Ph‐OSO_3_^−^	2.66	3.96 ± 0.11
CH_3_‐Ph‐OSO_3_^−^	2.68	4.30 ± 0.13
NO_2_‐Ph‐OSO_3_^−^	2.12	3.21 ± 0.09
CMR‐OSO_3_^−^	2.17	3.48 ± 0.10

#### 2.2.5 Peptides

A first reason for using peptides, besides classical thermometer ions, was to have a molecular class closer to some analytes of interest, i.e., with a higher number of degrees of freedom, and formed by protonation (in contrast to, e.g., benzylpyridinium ions, which are permanent cations). Leucine enkephalin (YGGFL, 228 degrees of freedom) is the most widely used thermometer peptide (Alexander and Boyd 1989; Goeringer, Asano, and McLuckey 1998). The first dissociation channel is the formation of the *b*_4_^+^ ion. The threshold energy (*E*_0_ = 1.19 ± 0.05 eV), ground state and transition state frequencies (allowing to calculate *k*(*E*) curves with the RRKM model, and thus *E*_app_) were re‐evaluated in 2010 (Sztaray et al. 2010). It is important to note, however, that most papers using YGGFL as thermometer ions for electrospray ionization sources predate this review (Drahos et al. 1999; Guo et al. 2003; Naban‐Maillet et al. 2005; Pak et al. 2008), and therefore the internal energy or characteristic temperatures values may suffer from a systematic bias.

Larger peptides such as LDIFSDF (351 DOF) and LDIFSDFR (420 DOF), with *E*_0_ values previously determined by time‐ and energy‐resolved surface‐induced dissociation (Bailey, Laskin, and Futrell 2003), were also used in internal energy and characteristic temperature determination in electrospray sources (Pak et al. 2008). Finally, the in‐source fragmentation of LPISASHpSpSKTR was studied by the Xia group in nano‐electrospray (Wang, Ouyang, and Xia 2010), although not for quantitative internal energy determination. Note that the experimental *E*_0_ values of cationized amino acids and small peptides have been determined by guided ion beam mass spectrometry (reviewed in (Armentrout 2023)). However, these were not used for ionization source characterizations.

## 3 Internal Energy in Electrospray Ionization: The Last 20 Years

### 3.1 Degrees of Freedom Effect

Naban‐Maillet et al. used MassKinetics besides the survival yield method to obtain the mean internal energies of ions <*E*_int_> in an ESI triple‐quadrupole instrument (Micromass Quatro I) (Naban‐Maillet et al. 2005). They compared the results obtained with thermometer ions of different nature and numbers of degrees of freedom: benzylpyridinium ions (69 < DOF < 81), protonated ester ions (72 < DOF < 84) and protonated leucine enkephalin (DOF = 228). The *E*_0_ values were obtained at the B3LYP/6‐31 G* level of theory, except for leucin enkephalin. At low collision energies, small molecules (69 < DOF < 228) had similar < *E*_int_ > , thus the charging process (protonation vs. permanent cations) and DOF of the molecules did not significantly influence their internal energy.

Later, the same group studied the internal energy distribution of larger peptide thermometer ions (228 < DOF < 441) (Pak et al. 2008): leucine enkephalin, LDIFSDF, LDIFSDFR, and RLDIFSDF. The mean internal energy increases linearly with the cone voltage, and the slope depends on the DOF of the ions. The intercept at 0 V on the cone is supposed to reflect the ionization contribution to the internal energy. The intercept was much lower for m‐CH_3_ BzPy and leucin enkephalin than for the larger peptides. Retrospectively, we conclude that the difference could be due either to differences in the ionization mechanism (charged residue mechanism for benzylpyridiniums *vs.* ion evaporation or chain ejection for larger peptides; see Section 3.5), or to biases in the *E*_0_ values used at the time. The *E*_0_ values of the benzylpyridinium ions used in MassKinetics were indeed underestimated compared to the values we can now recommend (Tables 1 and 2 herein).

### 3.2 Effect of the Desolvation and Ion Transfer Interface

The same ESI‐triple‐quadrupole mass spectrometer was used to evaluate the effect of the collision cell pressure on the CID spectra of leucine enkephalin. Three models (threshold, thermal and collisional) are implemented with MassKinetics, and the thermal model best reproduces the experimental data (Ichou et al. 2013), in line with the common practice to compare internal energy distributions to a “thermal‐like” Boltzmann distribution (Drahos and Vékey 1999). This assumption was contradicted initially by Rondeau et al. who found using an ESI source interfaced with a sector mass spectrometer that distributions were narrower than the thermal distribution (Rondeau et al. 2011). Of note, the average energies reported in that study were rather high, so the dissociation may be much faster than the energy exchange, and the internal energy distribution may be truncated at high energies. A later study by the same group considered the desolvation chamber of the ESI source as a collision area and used a partially elastic multiple collision model to account for the accumulation of internal energy in the ions. Here, the internal energy distribution of ions could be compared to a thermal distribution for characteristic temperatures in the range 1020–1550 K (Rondeau, Drahos, and Vékey 2014).

The gas flow regimes in the source can influence the internal energy distributions as well. Hampton et al. reported on the effect of an air amplifier setup, that focuses the ion beam and improves ion transmission, based on the Venturi effect (Hampton et al. 2008). Adding the air flow did slightly increase the average internal energy of electrosprayed ions, when produced from methanol solutions. The effect was even larger for ions produced with the AMUSE source (see Section 4).

Results obtained with different instruments can sometimes appear contradictory. Asakawa highlighted the impact of the capillary interface (capillary‐skimmer vs. capillary‐RF Lens) of an ESI‐triple quadrupole tandem mass spectrometer (Asakawa et al. 2023b) With a capillary‐skimmer interface, the internal energy distributions of ions are much lower and narrower (blue curve in Figure 1c) than with the capillary‐RF lens interfaces (yellow and red curves in Figure 1c). The effect is huge: the average internal energy can vary by a factor of two. In addition, the gas throughput into the first vacuum stage also influences the internal energy distribution of the ions (Asakawa et al. 2024). The density of molecules and atoms, present in ion transfer/focusing electrodes in the first vacuum stage, increased with the gas throughput. As a result, the ion's mean path in the first vacuum stage decreases, and the energy of ions decreases by decreasing the collision energy. The ion pressure in ESI did not affect the internal energy distribution of ions in this instrument configuration. These results highlight the relevance of performing experimental estimations of internal energies on each instrument, because the ionization step is only one contributor to the observed internal energy distribution.

In conclusion, the ion transmission devices between the source and mass analyzers influence the ion population's internal energy and in‐source fragmentation. Contemporary instruments increasingly use RF‐based ion guides including traveling wave ion guides and ion funnels. When used at high gas pressure, these ion guides can cool down the ion effective temperature close to the gas temperature (Tolmachev et al. 2004), even if higher internal temperatures were reached in the source region where the ions were produced. A quantitative example is given by Carpenter et al. , who deduced that benzylpyridinium ions reached ~650 K in the source, then were thermalized at 300 K in an RF hexapole (Carpenter et al. 2017). Note that traveling wave ion guides (Morsa, Gabelica, and De Pauw 2011, 2014a), differential mobility analyzers (Ieritano et al. 2020) and trapped ion mobility interfaces (Morsa et al. 2020b; Morsa et al. 2020c; Naylor et al. 2020) can also substantially heat the ions, but this subject will not be covered here.

### 3.3 Solvent Effects

Solvent effects on the ion internal energy in ESI were first evidenced with water/glycerol mixtures giving higher internal energies than water/methanol (Collette and De Pauw 1998). Rahrt et al. (2019) showed that the internal energy of ions produced by a ESI source with a quadrupole‐TOF hybrid mass spectrometer had been calculated by using five different benzhydrilpyridinium ions diluted in dichloromethane or methanol. The survival yield analysis led to internal energy distribution with maxima at 2.06 ± 0.13 eV in dichloromethane and 1.88 ± 0.11 eV in methanol, suggesting that the solvent effect had a small but significant influence. The higher evaporation enthalpy of methanol may enhance the evaporative colling.

The study by Bertrand et al. (2023) did not show a major influence of methanol versus acetonitrile on internal energies reached in electrospray, on the contrary to cold spray ionization (see Section 4). Hampton et al. (2008) also did not find a significant difference between electrospray from purely aqueous and 50:50 H_2_O:MeOH, in the absence of Venturi air amplifier. With the amplifier, the internal energy distribution was slightly higher in energy and narrower in the presence of 50% methanol, which could be ascribed to incomplete desolvation from purely aqueous solution (the Venturi effect accelerates the droplets, but does not cause enough turbulences for droplet desolvation). This contrasts with another study of the same group, without Venturi air amplifier and a different MS inlet, which showed lower internal energies in 50% methanol than in pure aqueous solutions (Harris et al. 2010). These examples illustrate the difficulty of drawing general conclusions from isolated small‐scale studies performed with specific source interfaces.

### 3.4 Comparison of Positive and Negative Ion Modes

Ion internal energy distributions *P(E)* had been determined in positive and negative ESI mode on a linear ion trap‐orbitrap hybrid mass spectrometer (Asakawa 2023a). H‐Ph‐OSO_3_^−^, CH_3_‐Ph‐OSO_3_^−^, NO_2_‐Ph‐OSO_3_^−^ and CMR‐Ph‐OSO_3_^−^ were diluted in water/acetonitrile (1:1). The kinetic shifts were considered. A supporting information figure compares the survival yields obtained using benzylpyridinium positive ions with the sigmoidal curves of the negative mode. The internal energies obtained in the two ionization modes are close, yet slightly lower in positive mode. Positive and negative thermometer ions can be considered as having the same internal energy distribution, as the small difference can be attributed to uncertainties in the RRKM calculations of the kinetic shift, or to the slightly different numbers of DOF.

### 3.5 Ion Production Mechanisms

In 2005, we hypothesized that if ions are expelled from the surface of the droplet at an early stage after droplet production, these ions may undergo more collisions and could end up with higher internal energies (Gabelica and Pauw 2005). The same conclusions are reached independently on ionic liquid electrospray emitters, used in the pure ionic regime (pure ion evaporation) for aerospace propulsion applications. By combining molecular modeling with retarding potential analysis of emitted ionic liquid clusters, Schroeder et al. deduced that ionic liquid dimers were evaporated with an internal temperature between 590 and 690 K, and trimers with a temperature between 990 and 1090 K (Schroeder et al. 2023).

Standard electrospray and nanospray are often defined in terms of flow rates (arbitrarily set at several µL/min for electrospray vs. in the nL/min range for nano‐electrospray). However, the source regime, a notion beautifully reviewed recently (Marginean 2024), could be relevant to the ion production regime and to internal energy differences. All high‐flow sprays requiring a nebulizing gas to aid droplet desolvation and gas‐phase ion production fall into the jetting regime, where the droplets are not well‐calibrated. In contrast, in nano‐capillary electrospray (static nano‐electrospray), a cone‐jet mode is established wherein the electrophoretic current suffices to sustain the production of monodisperse droplets charged close to the Rayleigh limit. When the emitter tips are narrow enough, each droplet contains on average one or zero analyte molecule, and ion suppression or adduct formation is not as prominent as in high‐flow electrospray (Juraschek, Dülcks, and Karas 1999; Juraschek et al. 1998; Susa, Xia, and Williams 2017). Droplet size and droplet initial charge might influence the ion internal energy: larger droplets may travel further down through the source interface and the analytes may be protected from collisions by a solvent shell. That holds if the ions are produced via a charged residue scenario from relatively large droplets.

The Zenobi group (Touboul, Jecklin, and Zenobi 2008) determined the internal energy distributions of ions produced by nano‐ESI, standard ESI, and sonic spray ionization (SSI). The latter does not use any voltage, and thus droplets are charged only to a low level (just statistically) and ions are supposedly produced only through a charge residue scenario. The internal energy distributions obtained with all three ionization methods were found overlap (the one with ESI is only slightly higher in energy). The authors concluded that benzylpyridinium ions in ESI and nano‐ESI are also produced via a charged residue scenario.

In contrast, specific nano‐electrospray effects were observed for peptides and proteins (Shepherd et al. 2024; Wang, Ouyang, and Xia 2010). First, the nanospray emitter position influences the voltage at which peptides dissociate or at which proteins unfold downstream the mass spectrometer, showing some degree of in‐source activation depending on the tip positioning (Shepherd et al. 2024). Even more intriguing, when nano‐electrospray is conducted in high conductivity and low‐flow rate conditions (not the conditions that provide the highest ion signal), peptide fragmentation occurred in the source (Wang, Ouyang, and Xia 2010). This was attributed to peptide ion emission directly from tiny first‐generation highly charged droplets, i.e., to an ion evaporation scenario. For peptides, the charged residue and ion evaporation mechanism compete (Aliyari and Konermann 2022), and the sequence determines the propensity to overcharging by ion evaporation or chain ejection (Xu et al. 2024). Thus, internal energy increase and fragmentation might indeed indicate the prevalence of an ion evaporation mechanism for some peptides.

In summary, source internal energy studies contradict the common intuition that small ions are produced via ion evaporation scenario whereas large ions are produced via the charged residue scenario: benzylpyridinium ions are smaller than peptides, but are produced via the charged residue mechanism, while peptides can be produced by ion evaporation or chain ejection. This could be at the origin of different characteristic temperatures observed for these two ion classes (Pak et al. 2008), although, as noted above, biases related to the *E*_0_ values used could also explain the difference. Further quantitative studies would thus be needed to shed a light on this matter. It would be interesting for example to study the interconnected effects of the emitter tip diameter, the electrothermal supercharging effect (Sterling et al. 2012), and supercharging (Iavarone and Williams 2003; Ogorzalek Loo, Lakshmanan, and Loo 2014) or stabilizing additives (Bagal et al. 2009; Sun, Kitova, and Klassen 2007), from an ion internal energy point of view. Studying pure ion emission at reduced pressure instead of at atmospheric pressure could also prove interesting.

## 4 Internal Energy Comparisons Between Atmospheric Pressure Ionization Methods

### 4.1 Methodology

We compiled the literature values reported with either benzylpyridinium or benzylammonium thermometer ions. We used average energy values written in the article. When the values were absent, but a figure of *P*(*E*) was given, we determined the <*E*> of the center of the distribution from the Figure. However, the values of <*E*> cannot be compared directly between articles because different levels of theory were used to obtain the *E*_0_ values, and most studies did not take the kinetic shift into account. To compare <*E*> values from articles using different thermometer ion series, we recalculated the <*E*> by taking the kinetic shift into account.

For benzylammonium ions, the values first reported by Donald's group (Stephens et al. 2015) (converted to eV) agree perfectly with values later recalculated by CCSD(T). We thus converted *E*_0_ to *E*_app_ with a reaction time τ = 10^−4^ s using

(1) $$E_{\text{app},\text{BzAm}}=1.506\times E_{0/\text{CCSD}(T)}\;–\;0.364,$$

a regression obtained from the values provided by (Asakawa and Saikusa 2022). For benzylpyridinium ions, when the *E*_0_ values had been obtained at the CCSD(T) level, the conversion was

(2) $$E_{\text{app},\text{BzPy}}=1.483\times E_{0/\text{CCSD}(T)}\;–\;0.272,$$

a regression obtained from the p‐OCH_3_, p‐CH_3_ and ‐H values (Carpenter et al. 2017) (the p‐NO_2_ values diverged from the linear regression). However, even up to recent years, most groups used the AM1 values reported back in 2004 (Gabelica, De Pauw, and Karas 2004a), without kinetic shift. For these data we used:

(3) $$E_{\text{app},\text{BzPy}}=2.66\times E_{0/\text{AM}1,2004}\;–\;1.594,$$

a regression obtained from the p‐OCH_3_, p‐CH_3_ and p‐NO_2_ ions.

**Figure 11:**
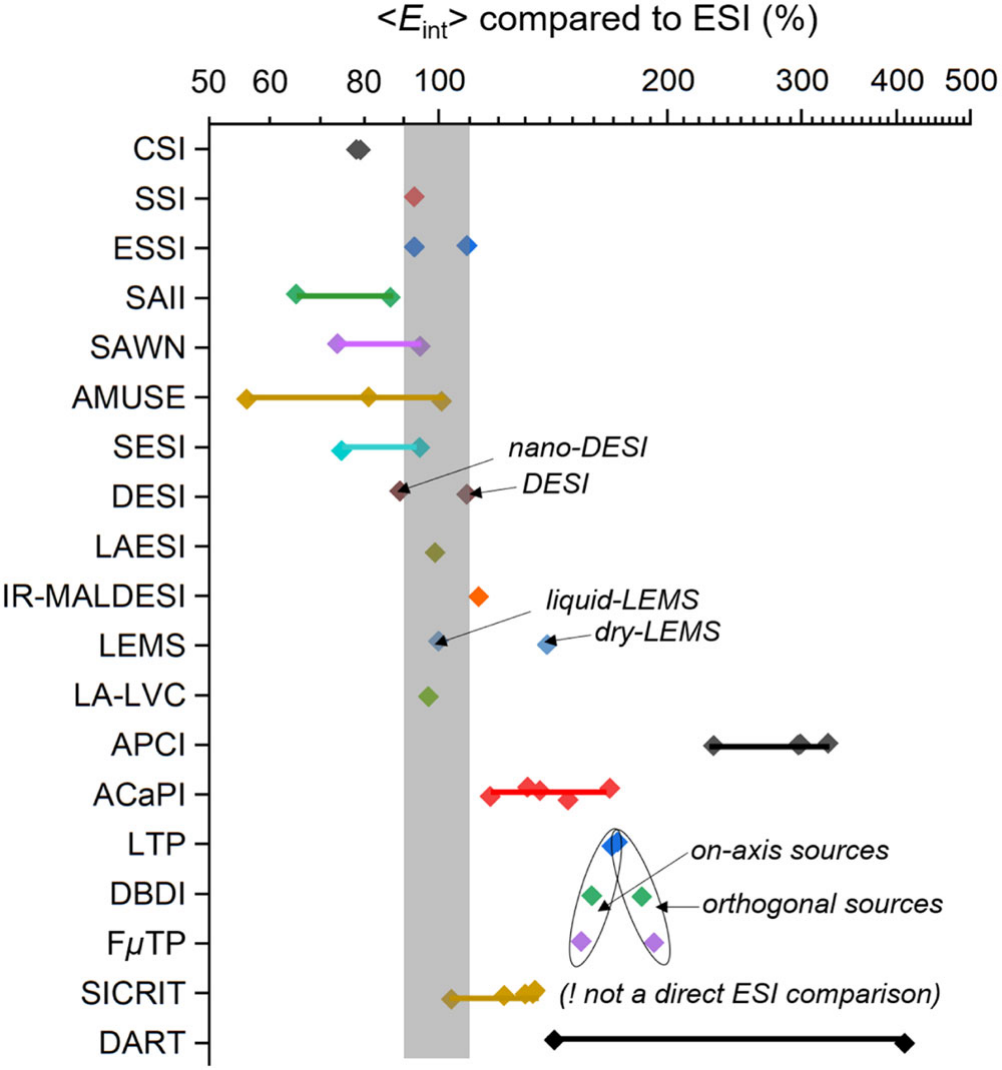
Comparison of relative mean internal energies obtained by the survival yield method, with those obtained with electrospray ionization in the same conditions (ESI: 100%. The log_10_ scale implies that “n times more” or “n times less” energy than electrospray has the same distance to 100%). The grey zone indicates a 10% variation compared to ESI; in this zone, the internal energy distributions largely overlap. [Color figure can be viewed at wileyonlinelibrary.com]

Yet comparing all sources in terms of <*E*> using *E*_app_ still does not guarantee proper comparison of what happens at the ionization level, because ion transfer influences the survival yield (see Section 3). We thus proceeded by comparison, using only data that compared <*E*_int_> values obtained with different ionization methods on the same MS interface, and divided the <*E*_int_> values obtained with that of ESI. The results are shown in Figure 11, which highlights ionization methods softer than ESI (> 90%), ionization methods giving similar energies to ESI (90%–110%), and ionization methods more energetic than ESI (> 110%).

### 4.2 Cold‐Spray Ionization (CSI)

Cold‐spray ionization (CSI) is a variant of electrospray ionization wherein the nebulizing gas is cooled to –40°C with liquid nitrogen (Sakamoto et al. 2000). CSI‐MS allows to characterize labile noncovalent complexes unobservable by ESI, to detect reaction intermediates, to uncover the solvated structure of organometallics compounds, or to understand the organization of deep eutectic solvents (Chen et al. 2020; Miras, Wilson, and Cronin 2009; Percevault et al. 2021; Yamaguchi 2013). Recently, the softness of CSI compared to ESI was evaluated quantitatively on an AccuTOF CS mass spectrometer equipped with a dual ESI/CSI source (Bertrand et al. 2023). The thermometer ions were substituted benzylpyridiniums using the survival yield method and MassKinetics. The thermometer ions were diluted in acetonitrile or methanol and a solvent effect was observed for CSI. The internal energies obtained in CSI are 79% and 73% those obtained in ESI with acetonitrile and methanol, respectively, while there is no significant difference between these solvents in ESI. In CSI, ions are cooler when produced from methanol than from acetonitrile. It can be linked to the surface tension (higher for acetonitrile than for methanol), although a larger solvent series would have been necessary to conclude on this. The reasoning is that, during the desolvation process, frictions between the charged droplets and the drying gas induce more heating with acetonitrile. This heating is then converted into thermal energy of the droplets and internal energy of the ions. In summary, in cold spray ionization, the droplets evaporate and disintegrate more slowly. The ions are protected better from collisions at the MS interface better than standard electrospray.

### 4.3 Methods With Droplet Formation Decoupled From Ion Production

Here we gather quantitative internal energy reports on sources where droplets are charged statistically instead of by applying a voltage to the emitter, and for which significantly lower average internal energies were observed than for ESI in the same interface and mass spectrometer settings. Other qualitative reports can be found on mechanospray ionization (Dugan and Bier 2022), Kelvin spray ionization (Özdemir et al. 2013), and vibrational sonic spray ionization (Li et al. 2021): these methods typically produce ions of lower charge states than electrospray, which favor the preservation of noncovalent complexes, and are thus thought to be softer than electrospray.

#### 4.3.1 Sonic Spray Ionization (SSI), Compared to Electrosonic Spray Ionization (ESSI)

Sonic spray ionization (SSI) uses a high nebulizing gas flow rate associate to a supersonic jet, and despite no voltage is applied to the capillary (Hirabayashi, Sakairi, and Koizumi 2002a, 2002b), droplets are charged statistically. ESSI is the same as SSI, but with a voltage applied to the capillary. The charge‐state distributions obtained with ESSI are narrower than in ESI (Takáts et al. 2004). The greater softness of these two techniques compared to ESI had been suggested by analyses of folded proteins, protein complexes, enzyme or protein‐ligand interactions (Hirabayashi, Sakairi, and Koizumi 2002b; Jecklin et al. 2011; Takáts et al. 2004; Wiseman et al. 2005). This softness was then assessed quantitatively and compared to ESI through the determination of internal energy distributions with the survival yield method using benzylpyridinium ions (Nefliu et al. 2008; Touboul, Jecklin, and Zenobi 2008). In the study of Nefliu et al. on a Thermo LTQ with a heated capillary inlet, the mean internal energy in ESSI was 109% of the one in ESI (Nefliu et al. 2008).

**Figure 12:**
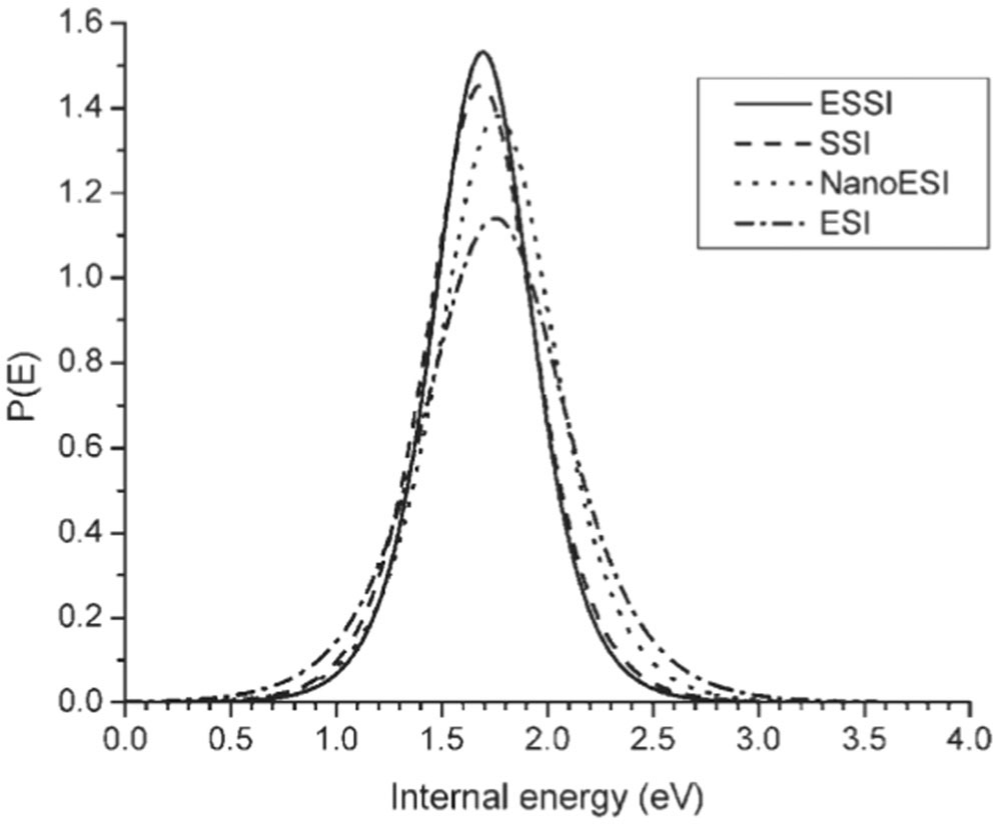
The internal energy distribution of para‐substituted benzylpyridium ions compared for different ionization techniques (ESI, nanoESI, ESSI and SSI), while keeping all instrumental parameters strictly constant. The curves are all very similar (within an experimental error of 2%), indicating that the ion formation mechanism must be the same for all spray techniques (Reproduced with permission from Touboul D, Jecklin MC, Zenobi R. Ion internal energy distributions validate the charge residue model for small molecule ion formation by spray methods. *Rapid Communications in Mass Spectrometry*. 2008;22(7):1062–1068. Copyright {2008} John Wiley and Sons.) (Touboul, Jecklin, and Zenobi 2008).

Touboul et al. studied benzylpyridinium salts diluted in water/methanol (50:50) on a hybrid quadrupole time‐of‐flight mass spectrometer equipped with a Waters Z‐spray interface (Touboul, Jecklin, and Zenobi 2008). The normalized internal energies obtained in SSI and ESSI are both equal to 93% of that of ESI, and the distributions were slightly narrower, more similar to nano‐ESI (Figure 12). We can see that a shift of 10% in the mean internal energy actually corresponds to quasi overlapping distributions. The authors thus considered that ESI, ESSI and SSI have intrinsically a similar ionization softness, but small differences could arise from the droplet sizes obtained in each setup. Touboul et al. highlighted the fact that in SSI, the gaseous ion formation can only be based on the charge residue model (CRM) because the formation of charged droplets is based on a statistical charging model, and each droplet contains few excess charges. Given that the shapes of the internal energy distributions were similar in SSI, ESSI and ESI, they concluded that the benzylpyridinium ion productions follows the charged residue scenario (Touboul, Jecklin, and Zenobi 2008).

#### 4.3.2 Solvent‐Assisted Ionization Inlet (SAII)

In solvent assisted ionization inlet (SAII), the liquid sample is introduced directly in a heated inlet tube, with no applied voltage. The ionization mechanism is based on a heat‐induced (statistical) charge separation at sub‐atmospheric pressure. The resulting desolvation process is then similar to the desolvation process in ESI. It was thus postulated that the internal energies of ions generated from these two ionization modes should be close (Pagnotti, Chubatyi, and McEwen 2011; Trimpin et al. 2012). A survival yield comparison between SAII and ESI was performed using substituted benzylpyridinium ions and an Orbitrap Exactive mass spectrometer (Fenner and McEwen 2015). The SAII was softer than the ESI, and the magnitude of the difference depends on the voltage applied to the tube lens and skimmer. The average internal energy obtained by SAII was 87% or 65% of that obtained by ESI, and the difference was greater at large skimmer voltages. This means that these voltages influence more the ion internal energies in ESI than SAII. The interpretation is that SAII droplets are less charged on average, take longer to desolvate, and therefore, ions are protected longer from direct in‐source collisional activation.

#### 4.3.3 Surface Acoustic Wave Nebulization (SAWN)

**Figure 13:**
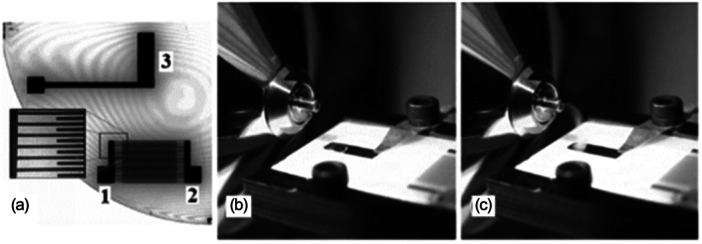
Surface acoustic wave nebulization process. (a) Configuration of the SAWN chip. Electrodes 1 and 2 are connected to a signal generator via a copper bin to produce the SAW. Electrode 3 is the link between the ground with the chip surface and the sample droplet. (b) Coupling between the SAWN and an atmospheric pressure ionization mass spectrometer. The droplet is observed on the SAWN chip. (c) Formation of a plume of liquid nebulized liquid entering the MS instrument (Reproduced with permission from Huang Y, Yoon SH, Heron SR, et al. Surface acoustic wave nebulization produces ions with lower internal energy than electrospray ionization. J Am Soc Mass Spectrom. 2012;23(6):1062–1070. Copyright {2012} American Chemical Society.) (Huang et al. 2012).

In surface acoustic wave nebulization (SAWN), surface acoustic waves are produced via a piezoelectric substrate, which will interact with the liquid analyte put on the substrate to generate little droplets (Figure 13). Analytes are nebulized as the droplets are charged statistically. The allure of the mass spectra is then similar to those obtained by ESI (Heron et al. 2010). The internal energy distribution of ions produced by SAWN has been investigated (Huang et al. 2012; Song et al. 2020). Huang et al. measured the survival yield of substituted benzylpyridinium ions, and results were compared with ESI (Huang et al. 2012). The experiments were performed with a LTQ mass spectrometer where ESI and SAWN sources were placed in front of the heated capillary inlet, with the same mass spectrometers settings.

SAWN is softer than the ESI: the normalized internal energy obtained by SAWN is 74% of that obtained in ESI. The magnitude of the difference depends on the capillary temperature and is greater at higher temperature. This suggests that ions are produced from droplets that desolvate faster in ESI than in SAWN, and thus that ions produced by ESI are exposed to heating in the capillary for more extended periods than in SAWN. In SAWN, the droplets are larger; the heating is dissipated in the desolvation process instead of being converted into excess internal energy.

#### 4.3.4 Array of Micromachined Ultrasonic Electrospray (AMUSE)

**Figure 14:**
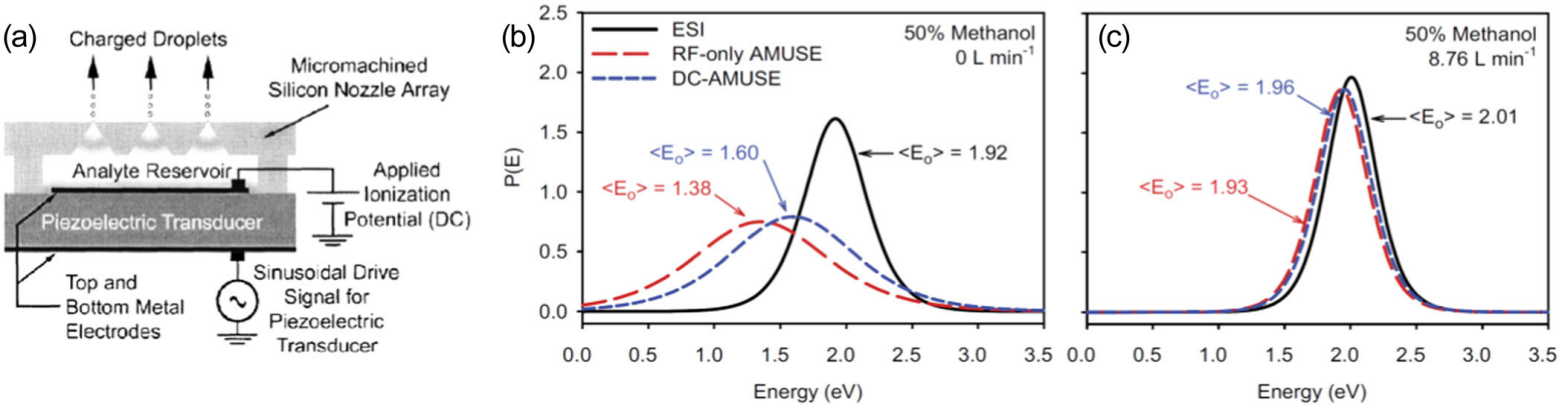
(a) the AMUSE setup (Adapted with permission from Aderogba S, Meacham JM, Degertekin FL, Federov AG, Fernandez FM. Nanoelectrospray ion generation for high‐throughput mass spectrometry using a micromachined ultrasonic ejector array. Appl Phys Lett. 2005;86:203110. Copyright {2005} AIP Publishing) (Aderogba et al. 2005). (b, c) Internal energy distributions (survival yield method, benzylpyridinium ions with AM1 2004 *E*_0_ values, no kinetic shift) obtained for ESI, RF‐only AMUSE and DC‐AMUSE in H_2_O:MeOH (50:50, v:v), (b) without the gas assistance and (c) with the assistance of a Venturi gas flow (Adapted with permission from Hampton CY, Silvestri CJ, Forbes TP, et al. Comparison of the internal energy deposition of Venturi‐assisted electrospray ionization and a Venturi‐assisted array of micromachined ultrasonic electrosprays (AMUSE). J Am Soc Mass Spectrom. 2008;19(9):1320–1329. Copyright {2008} American Chemical Society.) (Hampton et al. 2008). [Color figure can be viewed at wileyonlinelibrary.com]

In the Array of Micromachined UltraSonic Electrosprays (AMUSE) (Aderogba et al. 2005), the process of droplet formation and droplet charging are decoupled: an RF signal transmitted to a piezoelectric transducer. The ultrasonic wave in the sealed analyte reservoir forms a pressure gradient that emits droplets from the microarray (Figure 14a). Droplet charging is further induced by applying a DC potential difference between the array and the mass spectrometer entrance.

Hampton et al. compared the internal energy distributions obtained with RF‐only AMUSE (statistical droplet charging), DC‐AMUSE (droplet polarity enrichment) and regular ESI, in front of the heated capillary inlet of a Thermo LTQ mass spectrometer (Hampton et al. 2008). They also compared the measurements in the absence or in presence of an air amplifier based on Venturi gas flow, which increases the ion signal. RF‐only AMUSE was the softest ionization method, followed by DC‐AMUSE, both softer than ESI (Figure 14b). However, Venturi air flow assistance abolished these differences: the internal energy distributions are all slightly higher in energy and are now almost superimposed (Figure 14c).

#### 4.3.5 Summary

These results highlight one caveat of the comparison studies: the differences in softness due specifically to the ionization method can be evidenced only with the softest possible ion transfer interfaces, i.e., in conditions different from those giving the best ion signal. Like SSI, SAWN, SAII and RF‐only AMUSE all operate without voltage, and the droplets can only be charged statistically. The fact that the study on SSI, ESSI and ESI failed to detect significant differences in internal energy distributions (Touboul, Jecklin, and Zenobi 2008) could also be due to heating in the source interface, causing sufficient droplet desolvation and ion production for all sources, and thermalization of the ion population to the same level.

### 4.4 Ambient Ionization Methods Involving Analyte Pick‐Up by Electrospray Droplets

#### 4.4.1 Secondary Electrospray Ionization (SESI)

**Figure 15:**
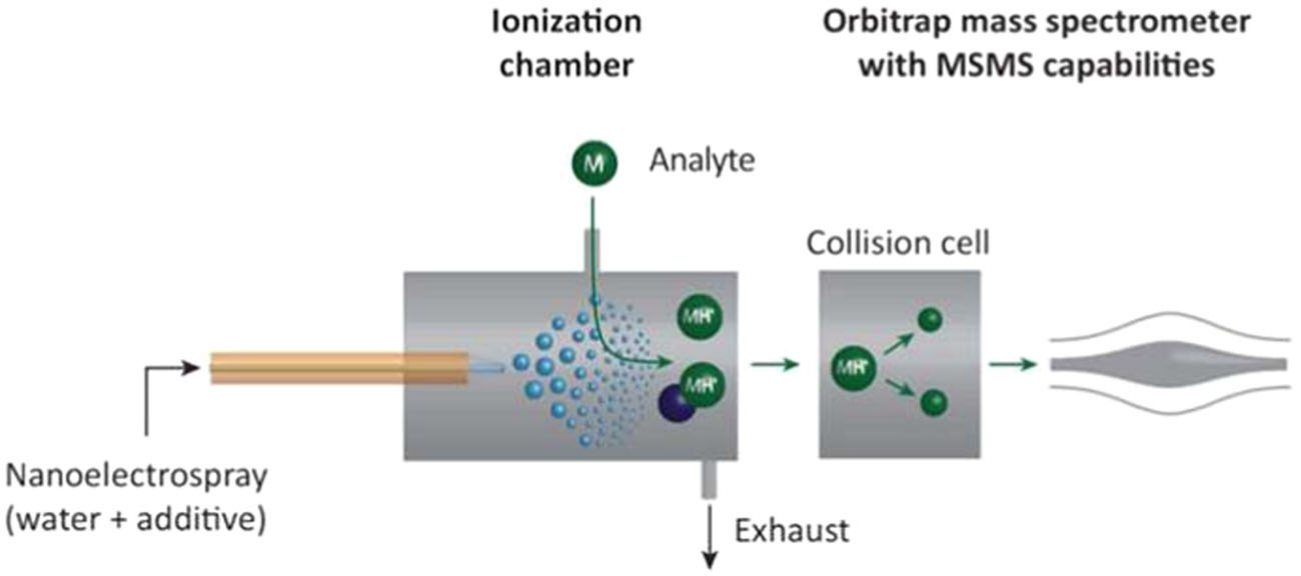
Schematic representation of secondary electrospray ionization (SESI) (Adapted with permission from Bruderer T, Gaisl T, Gaugg MT, et al. On‐Line Analysis of Exhaled Breath Focus Review. Chem Rev. 2019;119(19):10803‐10828. Copyright {2019} American Chemical Society.) (Bruderer et al. 2019). [Color figure can be viewed at wileyonlinelibrary.com]

Secondary electrospray ionization mass spectrometry was developed for untargeted metabolomics analyses such as online breath analysis (Bruderer et al. 2019). SESI is a variant of ESI based on the observation that, during ESI, compounds present in the ionization region can be also ionized. A secondary ionization can be induced when the primary ions formed by ESI are brought into contact with the gas‐phase molecules. Figure 15 illustrates the principle of SESI‐MS. The spray formed by nano‐ESI is composed of additives to increase the conductivity and generate primary ions. Collisions between these primary ions and the gaseous analytes lead to proton‐transfer reactions.

SESI was presumed to be a soft technique because for various chemical classes, the molecular ions is predominantly observed (Martinez‐Lozano Sinues, Criado, and Vidal 2012; Vidal‐de‐Miguel and Herrero 2012). Kaeslin et al. quantitatively evaluated the softness degree of SESI via the survival yield method (Kaeslin et al. 2022). They used a Q Exactive Plus mass spectrometer with two different ion sources: a standard ESI source and a SESI source. For SESI, the benzylamines were dissolved in ACN and their headspace vapors were brought in contact with the electrospray plume inside the SESI source. Then both for ESI and SESI, the electrospray liquids were H_2_O + 0.1% formic acid. The normalized internal energies calculated depend on the soft tuning: average internal energies in SESI were 75% of those in ESI in the softest source conditions (low transfer capillary and low S lens RF voltage) but only 95% of those in ESI in usual conditions. The authors interpret the results in terms of ion embedding in the protective shell of a droplet for most of their trajectory in the source and are thus less subjected to collision activation.

#### 4.4.2 Desorption Electrospray Ionization (DESI)

**Figure 16:**
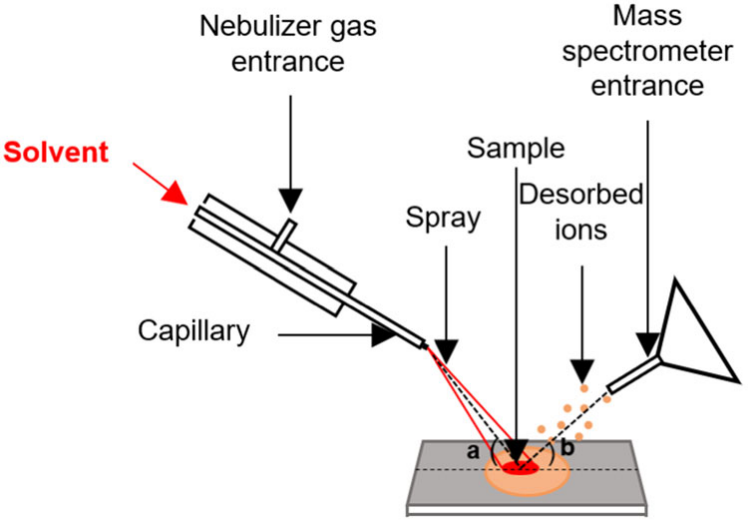
Configuration of a desorption electrospray ionization (DESI) source. [Color figure can be viewed at wileyonlinelibrary.com]

Desorption electrospray ionization (DESI), developed first by the Cooks group (Cooks et al. 2006; Takáts, Wiseman, and Cooks 2005; Takáts et al. 2004), is a variant of ESI where solid samples are analyzed by spraying charged solvent droplets onto them (see Figure 16). The ESI droplets pick up analytes from the surface.

Nefliu et al. compared the internal energy distribution of ion generated by DESI, ESI and ESSI (Nefliu et al. 2008), using the survival yield method with benzylpyridinium cations. With optimal settings defined as a tube lens voltage of 100 V and a capillary voltage ranged between 70 and 100 V, similar shapes of internal energy distribution were obtained. The different droplet history in ESI, ESSI and DESI had thus only a limited effect on the internal energy distribution (<*E*_int_> in DESI was 109% of that in ESI), indicating that the final desolvation stage of the ionization process (ion evaporation or charge residue mechanism) is similar for the three ionization methods. The authors also tested the effect of the nebulizing gas pressure, the solvent flow rate and the distance from the sprayer tip or surface impact spot to the MS inlet. The parameters related to the droplet flux were less important than those related to the MS interface.

The effect of nonaqueous spray solvents on the internal energy of ions generated from DESI was also investigated (Badu‐Tawiah et al. 2010). The internal energy of ions generated by a nonaqueous solvent (CHCl_3_/THF (50/50)) was lower than from an aqueous solvent such as MeOH/H_2_O (50/50). During the desolvation process, volatile spray solvents can dissipate heat by evaporation. The surface temperature of the droplet decreases and then the internal energy of ions decreases.

Hartmanova et al. investigated the internal energy of ions generated by nano‐DESI, a variant of DESI wherein the emitter diameter is smaller (Hartmanová et al. 2014). The mean internal energy of ions generated by nano DESI was 89% of that obtained in ESI. The authors suggest that the two source designs may affect how droplets are transferred through the heated capillary inlet. Also, the distributions overlap to a significant extent. Given the discrepancy of results between DESI (appearing slightly more energetic than ESI) and nano‐DESI (appearing slightly less energetic than ESI), we conclude that variations of <*E*_int_> in the order of 10% are largely within uncertainty due to different droplet size regimes, and thus that DESI has similar softness as ESI.

#### 4.4.3 Electrospray Pick‐Up of Laser‐Desorbed Analytes

We will not cover here all desorption/ionization sources (some recent contributions about internal energies can be found in the following references: (Fu et al. 2019; Luo et al. 2006; Milasinovic et al. 2014; Shea et al. 2007; Woods, Miller, and Baer 2003)), but only those involving laser desorption at atmospheric pressure followed by pick‐up by electrospray droplets. The following sources were directly compared with ESI: atmospheric‐pressure mid‐infrared laser ablation ESI (LAESI) (Nemes, Huang, and Vertes 2012), infrared matrix‐assisted laser desorption/ionization (IR‐MALDESI) (Tu and Muddiman 2019), femtosecond 800‐nm laser ESI (LEMS) (Flanigan et al. 2014), and 355‐nm UV laser ablation‐liquid vortex capture ESI (LA‐LVC) (Cahill et al. 2015). In brief, the survival yield method confirms that the softness of these methods is like that of ESI (from 97% to 113%, always with largely overlapping distributions). The only exception is when femtosecond 800‐nm laser ESI is performed on dried benzylpyridinium salts (dry‐LEMS, 139%) instead of on wetted samples (liquid‐LEMS, same as ESI).

### 4.5 Electric Discharge or Plasma‐Based Sources

**Figure 17:**
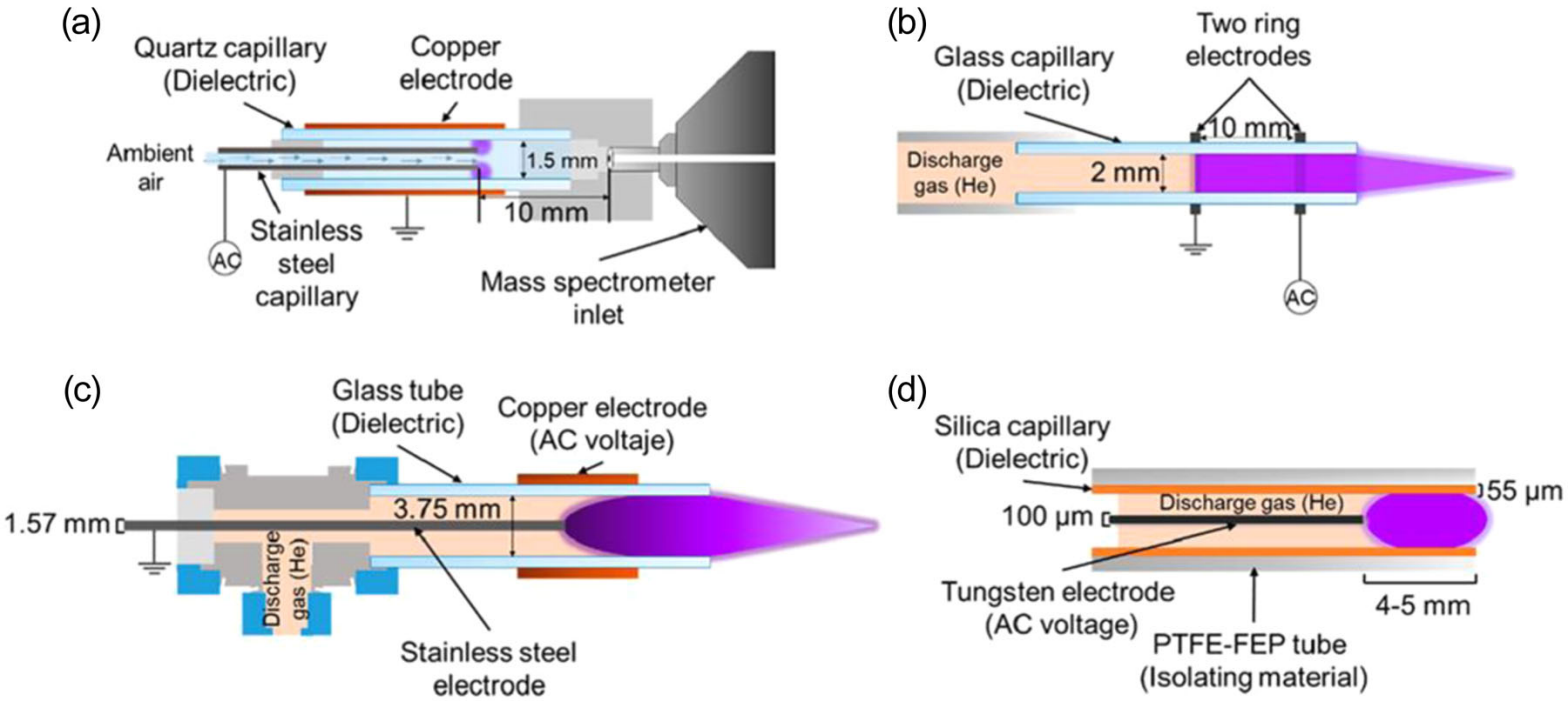
Diagrams of the dielectric barrier discharge ion sources: (a) active capillary plasma ionization (ACaPI), (b) dielectric barrier discharge ionization (DBDI), (c) low temperature plasma (LTP), and (d) flexible microtube plasma (FμTP) (Bouza et al. 2023). [Color figure can be viewed at wileyonlinelibrary.com]

Plasma‐based sources are ionization methods using electric discharges or plasmas to produce charged species (Smoluch, Mielczarek, and Silberring 2016a). The best‐known source using this technology is atmospheric pressure chemical ionization (APCI). The principle of this atmospheric pressure ionization method is the formation of a spray, assisted by strong gas flow and high temperature, followed by an electric discharge to produce ions. Other sources emerged based on the same concept, but using a plasma to aid ionization, such as flexible microtube plasma (FµTP), dielectric barrier discharges ionization (DBDI), low‐temperature plasma (LTP) and the active capillary plasma ionization (ACaPI), developed by the Zenobi group (Nudnova, Zhu, and Zenobi 2012). The principle of these Dielectric Barrier Discharges (DBD) sources is to apply a high voltage alternating waveform between two electrodes separated by an insulator. It results in a plasma, which characteristics depend on the operating pressure, geometric configuration, size and density (Niu et al. 2021). The main differences between source configurations are summarized in Figure 17. A great advantage of these sources is their possible coupling to GC‐MS and the ionization of nonpolar compounds (Ayala‐Cabrera et al. 2023; Mirabelli, Wolf, and Zenobi 2017).

#### 4.5.1 Atmospheric Pressure Chemical Ionization (APCI)

Donald's group evaluated the internal energies of ions generated through a DBD‐based ion source using substituted benzylammonium ions (Bouza et al. 2023; Dumlao, Khairallah, and Donald 2017a; Stephens et al. 2015). The mass spectrometer was a linear quadrupole ion trap mass spectrometer. A first study compared the influence of LTP and APCI sources on the ion's internal energies (Stephens et al. 2015). With a 35 V in‐source CID and a capillary temperature of 250°C, LTP was significantly softer than APCI. ESI was not included in the comparison, but LTP was compared to ESI in a subsequent study (Bouza et al. 2023). We thus deduced stepwise that the average internal energy in APCI was from 230% (at 150°C) to 330% (at 250°C) of that in ESI at 250°C.

#### 4.5.2 Dielectric Barrier Discharge (DBD) Sources

Vibrational and rotational temperatures can be determined directly from the rovibrational spectra of small ions produced in these sources. For example, the rotational temperature of a flowing atmospheric‐pressure afterglow (FAPA) source was estimated around 1100 K (Shelley, Chan, and Hieftje 2012). For a home‐made DBD source, the rotational temperature was 300 K while the vibrational temperature was 5000 K (Furter and Hauser 2018).

In terms of comparisons with ESI, the internal energy distributions obtained with the four DBD‐based ion source presented above (Bouza et al. 2023). The normalized internal energies obtained for DBDI, LTP, FµTP and ACaPI are respectively 185%, 172%, 192% and 117% of those in ESI. Parameters such as the distance between the DBD plasma jet and the source, as well as the capillary nature, strongly influence the ion fragmentation yields. The largest effect came from the positioning of the plasma device: placing the device on‐axis resulted in a decrease of internal energy for DBDI, LTP, and FµTP to ~160% of that in ESI.

Remarkably, ACaPI is the softest DBD‐based ion source, barely more energetic than ESI (117% in the softest settings). ACaPI is already on‐axis, and the internal energy distribution does not change according to the shape of the waveforms (Dumlao et al. 2017b). To maximize softness, it is important that ions in the ACaPI source pass through the center of the ring (Gyr et al. 2019), “like a lion in the ring of fire” (Bouza et al. 2023). If ions pass through the halo of the plasma rather than in the center, or if the voltage is increased, the internal energy in ACaPI increases up to ~170% of that in ESI.

Some readers might be interested in the SICRIT device. The brand name itself suggests SICRIT imparts little internal energy. SICRIT is a variant of ACaPI. Unfortunately, the publication reporting internal energy distributions in SICRIT (Huba, Mirabelli, and Zenobi 2019) did not include a direct comparison with electrospray on the same mass spectrometer. We thus made the gross approximation that the MS tuning approach in the SICRIT study would be similar to those in the SESI study (Kaeslin et al. 2022), also by the Zenobi group on benzylammonium ions, despite that different mass spectrometers were used. With this caveat in mind, SICRIT was found to impart internal energies only slightly higher than ESI (120% to 130% with dry gases, and similar to the ESI study when adding humidity to the N_2_ gas stream). These estimations agree with the ACaPI results and confirm that SICRIT is nearly as soft as ESI. Further studies with both sources on the same interface, and interface tuning to the softest possible transfer conditions, would be required for better quantitative comparisons.

#### 4.5.3 Direct Analysis in Real Time (DART)

**Figure 18:**
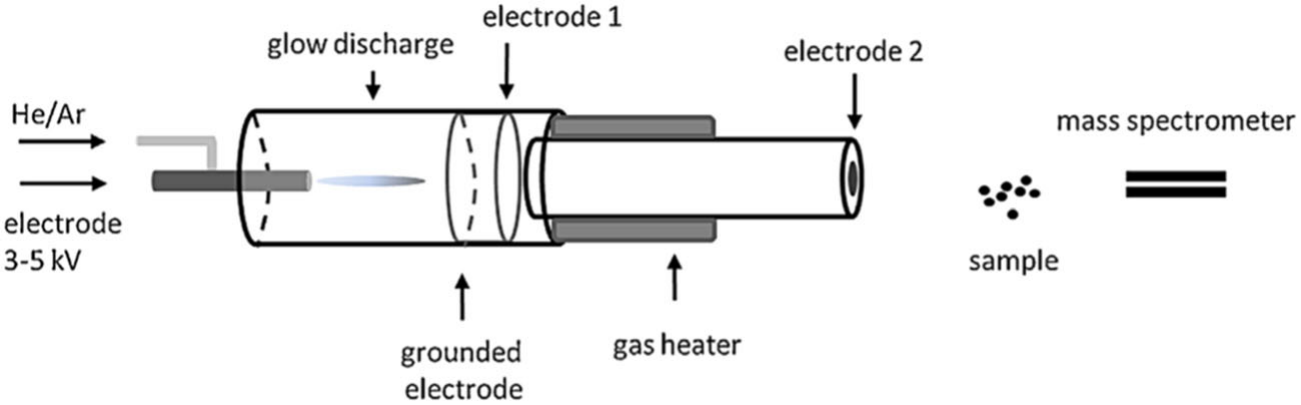
Schematic representation of DART source (Reproduced with permission from Smoluch M, Mielczarek P, Silberring J. Plasma‐based ambient ionization mass spectrometry in bioanalytical sciences. Mass Spectrom Rev. 2016b;35(1):22–34. Copyright {2016} American Chemical Society.) (Smoluch, Mielczarek, and Silberring 2016b). [Color figure can be viewed at wileyonlinelibrary.com]

DART is an ambient MS ionization technique developed by JEOL (Cody, Laramée, and Durst 2005). The ionization process is divided into two steps (Figure 18). The gas plasma is formed where the gas (helium, argon or nitrogen) enters a discharge chamber and comes into contact with a discharge needle (typically 1–5 kV). Then, the hot plasma gas is transferred to the ion source where it interacts with the sample and transfers its charges to the analyte molecules. Several ionization processes can occur in the DART source, such as Penning ionization, proton transfer or charge transfer (Gross 2014; Guo et al. 2017; Harris et al. 2010).

Two articles investigated the internal energies of ions generated by DART‐MS (Dumlao, Khairallah, and Donald 2017a; Harris et al. 2010). Harris et al. calculated the internal energy distributions of substituted benzylpyridinium diluted in nanopure water (Harris et al. 2010). The mass spectrometer used was a commercial DART ionization source and a JMS‐100TLC orthogonal acceleration time‐of‐flight. The mean internal energy obtained with DART was 142% of that obtained with the ESI source. The thermal contribution was higher in DART than ESI. That same conclusions are reached in the work of Dumlao et al. where the internal energies of substituted benzylammonium are estimated by using a linear quadrupole ion‐trap mass spectrometer (Dumlao, Khairallah, and Donald 2017a). However, here the mean internal energy in DART was 410% of that obtained in ESI on the same instrument. The modes of operation of DART are very diverse, but this ionization source is clearly more energetic than ESI. Humidity also decreases fragmentation in DART (Newsome, Ackerman, and Johnson 2016).

## 5 Conclusions

Sources wherein droplet formation is decoupled from electrophoretic charging (e.g., SAII, AMUSE or SAWN) can be softer than electrospray sources. However, no major differences in internal energies were found for ions produced from different variants of ESI, except for cold spray ionization (CSI). Among methods wherein the analytes are collected by ESI droplets, secondary electrospray ionization (SESI) is the softest, while desorption electrospray ionization (DESI) or electrospray of laser‐desorbed analytes are energetically similar to electrospray. Finally, although plasma and discharge‐based sources such as atmospheric pressure chemical ionization (APCI) or direct analysis in real time (DART) are more energetic than electrospray, new variants of dielectric barrier discharge sources, such as ACaPI and its commercial variant SICRIT, are capable to ionize gas‐phase molecules while conveying barely more internal energy than ESI.

It was, however, difficult to draw general conclusions by piecing together all small‐scale studies reviewed here. One reason is that almost each study is performed with a different source interface. Probing the outcome of the electrospray process in the mass analyzer is like trying to deduce the entire movie plot from just the last scene. Droplet size and desolvation history matter enormously because ions that traverse the harshest parts of the transfer interface while being protected by a solvent shell, which dissipates internal energy upon evaporation, will end up with less internal energy than those ejected “naked” early in the source.

Ions produced via the charged residue mechanism have less internal energy than ions produced by ion evaporation right after droplet formation. Sources that decouple droplet formation from droplet charging, and that charge the droplets less on average, are thus also more favorable for native MS applications: the preservation of the noncovalent complexes is favored by keeping the solvent around as late as possible and by minimizing Coulomb repulsion. Future source developments aimed at maximizing large ion production from lowly charged droplets should benefit the field of structural biophysics. However, characterizing the internal energy of ions produced in conditions adequate for desolvating macro‐ions will require larger thermometer ions. The GIBMS measurement of *E*_0_ values requires well‐controlled single‐collision conditions, yet attaining dissociation, which becomes increasingly difficult as the size of the ions increases because of the magnitude of the kinetic shift. Determining their *E*_0_ values experimentally for ions larger than tripeptides will require new approaches, such as modeling of collisional heating and cooling combined with ion trajectory calculations (Donor, Shepherd, and Prell 2020; Hoxha et al. 2001; Prell 2024). The availability of more massive computer power and new functionals will certainly help to calculate *E*_0_ values for thermometer ions with a larger number of DOFs, to calibrate instrumental conditions relevant to molecules of wider ion mobility and *m/z* ranges.

As for small molecule analytes, in‐source fragmentation effects are of paramount importance for correct data interpretation. Think about problems arising with database searches or machine learning algorithms are trained on datasets recorded with different activation conditions. Although several authors proposed to use thermometer ions to monitor, standardize and control in‐source fragmentation (Bristow et al. 2002; Hopley et al. 2008; Lecchi et al. 2009), they were probably right too soon, and their recommendations were not acted upon. The urgency is however now recognized, and in the wake of recent efforts to standardize MS/MS activation conditions (Hoang et al. 2024), it is urgent to tackle the problem of internal energies and fragmentation at the interfaces of supposedly “soft” ionization sources.

To do so, we would need further thermometer ions, with a chemical nature more relevant to the applications. Benzylamine metabolites and peptides are a good start, but fragile lipids, oligonucleotides and large peptides/small proteins or model polymers would be useful. Currently, multiply charged thermometer ions, and large thermometer ions with sufficiently low *E*_0_ values are in need. Efforts to develop widely applicable internal energy calibrants are under way in our laboratory.

## Author Contributions

**Emilie Bertrand:** data curation, formal analysis, investigation, methodology, writing–original draft and editing. **Valérie Gabelica:** conceptualization, data curation, investigation, methodology, project administration, supervision, visualization, writing–original draft, writing–review and editing.
